# Is sarcopenia a real concern in ankylosing spondylitis? A systematic literature review

**DOI:** 10.1007/s41999-024-00968-1

**Published:** 2024-04-03

**Authors:** Chiara Ceolin, Mario Virgilio Papa, Laura Scagnellato, Andrea Doria, Giuseppe Sergi, Roberta Ramonda

**Affiliations:** 1 Department of Medicine (DIMED), Geriatrics Unit, University of Padova, Via Giustiniani 2, 35128 Padua, Italy; 2 Department of Medicine (DIMED), Rheumatology Unit, University of Padova, Via Giustiniani, 2, 35128 Padua, Italy; 3 Radiology Unit, University of Padova, Via Giustiniani 2, 35128 Padua, Italy

**Keywords:** Sarcopenia, Ankylosing spondylitis, Axial spondyloarthritis, Muscle mass, Muscle strength

## Abstract

**Purpose:**

Sarcopenia is a condition defined as loss of muscle mass and strength, associated with poor functional performance and disability. Sarcopenia can be exacerbated or worsened in presence of inflammation, sedentary lifestyle and cytokine imbalance, thus it frequently occurs in people affected by rheumatic diseases. This systematic literature review aims to explore the association between sarcopenia and spondyloarthritis (SpA) and its most frequent manifestation, i.e. ankylosing spondylitis (AS).

**Methods:**

The Scopus, PubMed, and Web of Science databases were searched for articles on muscle mass, muscle strength and axial SpA, from any date to November 2023. Only studies written in English were considered. The methodological quality of the studies included in the review was evaluated using the Newcastle–Ottawa Scales for observational studies and for case–control studies.

**Results:**

190 papers were retrieved from the searches, 14 of which met the inclusion criteria. Rather than diagnosis of sarcopenia, pre-sarcopenia or probable sarcopenia were frequent in people with AS, with a great reduction especially of muscle strength. The pre-sarcopenia status appears to be related to high AS disease activity, suggesting that chronic inflammation resulting in pain, less movement and decreased physical activity could play a role in the muscle heath of AS patients.

**Conclusions:**

Our review confirms the existence of an association between AS and loss of muscle strength—likely sarcopenia—already at a young age. Preventive and early strategies should be adopted to ensure successful aging for individuals with AS.

**Supplementary Information:**

The online version contains supplementary material available at 10.1007/s41999-024-00968-1.

## Key summary points

**Aim:**

Explore the association between sarcopenia and spondyloarthritis (SpA), particularly ankylosing spondylitis (AS), with a focus on muscle mass, strength, and axial SpA.

**Findings:**

The occurrence of pre-sarcopenia or probable sarcopenia was more prevalent than sarcopenia, particularly marked by a significant reduction in muscle strength. The association of pre-sarcopenia with elevated AS disease activity suggests a potential influence of chronic inflammation on muscle health.

**Message:**

Evidence points to a correlation between AS and premature muscle strength loss, suggesting a potential onset of sarcopenia, underscoring the importance of early intervention strategies for successful aging in individuals with AS.

**Supplementary Information:**

The online version contains supplementary material available at 10.1007/s41999-024-00968-1.

## Introduction

Ankylosing spondylitis (AS) is an inflammatory disease categorized under seronegative spondyloarthritis (SpA). This condition typically manifests in individuals aged 20–45, primarily affecting axial regions, but can also involve peripheral joints and enthesis with symptoms like synovitis, enthesitis, and dactylitis [1]. Common early symptoms include inflammatory back pain and characteristic stiffness exacerbated by inactivity [1]. Extra-articular manifestations such as uveitis, psoriasis, mucositis, and chronic inflammatory bowel disease may also occur [1]. Chronic pain and joint dysfunction in AS lead to a more sedentary lifestyle, which worsens with acute inflammation [1, 2]. Recent research indicates a molecular connection between muscle, joints, entheseal tissues, and bone health with the Interleukin 23 (IL23) and Transforming Growth Factor beta (TGFbeta) pathways potentially playing a major role in musculoskeletal tissue changes [3].

By the same mechanisms, poor physical activity and chronic inflammation induce chronic complications, such as osteoporosis [4]. In the last decades, there has been much debate as to whether sarcopenia could also be a complication associated with AS [5]. Sarcopenia is defined according to the European working group on sarcopenia in older people (EWGSOP) criteria as an abnormally low muscle mass associated with low skeletal muscle strength and/or poor physical performance, leading to an increased risk of unfavorable outcomes such as physical disability and poor quality of life [6]. Primary sarcopenia usually is associated with older age, as it is linked to physiological muscle aging [7]. Considering the pathophysiology of SpA, we would expect an increased incidence of secondary sarcopenia in patients with AS, especially in the presence of active disease activity. Despite these considerations, current evidence fails to establish the exact prevalence of sarcopenia in AS, possibly due to heterogeneous studies and varying definitions of sarcopenia.

Therefore, this systematic literature review aims to identify accessible studies examining poor muscle health associated with AS. The goal is to critically appraise these studies in light of the latest hypothesis on AS pathophysiology and reevaluate the potential role of low muscle mass and/or strength in exacerbating AS. Recognizing decreased muscle health in AS patients should prompt preemptive and therapeutic, system-specific interventions to improve function and quality of life.

## Methods

### Eligibility criteria

Inclusion criteria were: (a) studies investigating the relationship between muscle mass and muscle strength, and axial SpA; (b) studies that compared patients affected by axial SpA to healthy controls or different rheumatic disease; (c) population aged ≥ 18 years; (d) defined and validated criteria to assess the presence of sarcopenia; and (e) published studies on the topic of interest using any study methodology, with a focus primarily on case–control, prospective, and cross-sectional studies; (f) availability of full text of the original research paper.

Exclusion criteria were: (a) case reports, abstracts, letters, and editorials; (b) studies not written in English; (c) animal model studies; (d) articles focusing on the therapeutic effect on muscle health of any type of drug (such as biotechnological drugs).

### Information sources

The Scopus, PubMed, and Web of Science databases were searched from any date to November 2023.

### Search strategy

The databases were searched for the terms “ankylosing spondylitis”, “axial spondyloarthritis”, “sarcopenia”, “cachexia” and “muscle strength”. The detailed search string utilized for bibliographic retrieval is provided in the supplementary materials.

### Selection and data collection process

This review adheres to Preferred Reporting Items for Systematic Reviews and Meta-Analyses (PRISMA—http://www.prisma-statement.org/) and Meta-analysis of Observational Studies in Epidemiology guidelines [8]. References cited in the selected papers were examined to identify any other potential articles. After selection of papers by a first reviewer (C.C.), the whole process was repeated and confirmed by a second reviewer (M.V.P.) to ensure validity of inclusion. Differences of opinion were discussed until consensus on inclusion or exclusion was reached with a third reviewer (L.S.). The Rayyan software, a web-based tool tailored for systematic review coordination, was utilized to expedite the screening phase. The methodological quality of each article selected for inclusion in the review was assessed by two reviewers (C.C., M.V.P) using the Newcastle–Ottawa Scales (NOS) for observational studies and for case–control studies.

### Data extraction

Titles and abstracts of selected articles were screened for relevance. The following data were extracted: (1) study design; (2) sample size, including number of female patients, number of cases and controls; (3) median/mean age of participants; (4) body composition assessment tool; (5) muscle strength assessment tool; (6) pre-sarcopenia/sarcopenia assessment tool; (7) axial SpA duration; (8) axial SpA disease activity assessment tool and mean/median value; (9) outcome on muscle mass; (10) outcome on muscle strength; (11) prevalence of pre-sarcopenia/sarcopenia; (12) possible correlation with disease activity; and (13) study limitations.

## Results

**Fig. 1:**
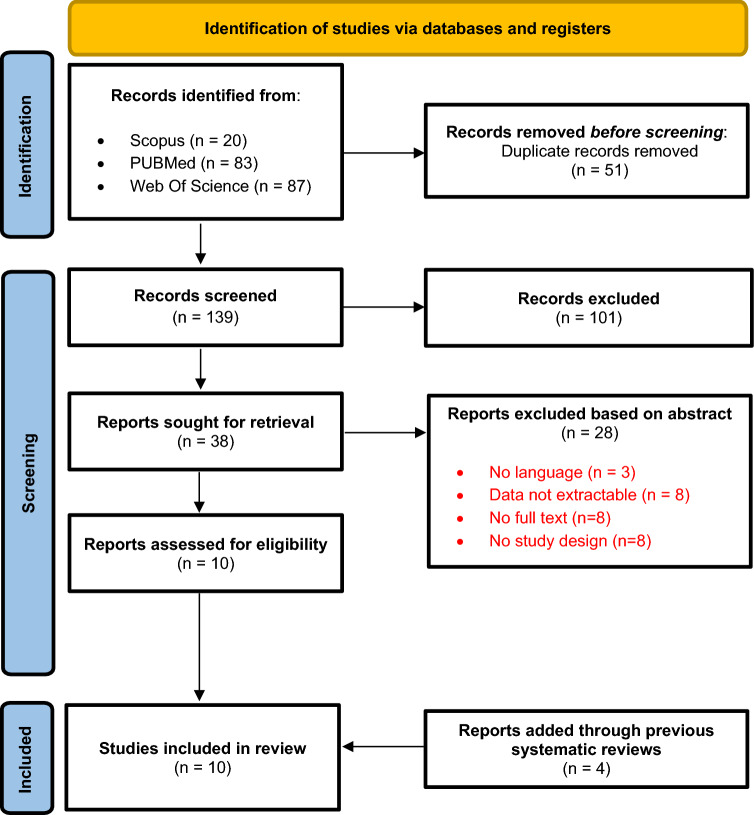
PRISMA flow diagram of the process of (and reasons for) including and excluding studies

A total of 190 studies were identified from the database searches, of which 51 duplicates were excluded. After reviewing the titles and abstracts, 101 records were discarded leaving 38 papers whose full manuscripts were examined in detail. A further 28 papers were discarded as ineligible, leaving 10 studies for full appraisal. Four additional records were added through a previous systematic review (Fig. 1). NOS scores of the included articles are shown in Tables 1 and 2. After evaluation by two researchers, the studies received an average NOS score of 6.0, indicative of good quality.

**Table 1:** Study quality assessment using Newcastle–Ottawa scale for observational studies

References	Selection				Comparability (matched analysis)	Assessment of outcome	Outcomes	Adequacy of follow-up of cohorts	NOS score
	Consecutive or obviously representative series of cases	Representativeness of exposed cohort	Ascertainment of exposure	Demonstration that outcome of interest was not present at the start of study			Follow up long enough for the outcome		
Barone [21]	*	*	*	–	**	*	–	–	6
Demirkapi [9]	–	*	*	–	**	*	–	–	5
Kanjanavaikoon [22]	–	**	*	–	–	**	–	–	5

*NOS* Newcastle Ottawa quality assessment scale

**Table 2:** Study quality assessment using Newcastle–Ottawa scale for case–control studies

References	Selection				Comparability of cohorts	Ascertainment of exposure	Outcomes	Non response rate	NOS score
	Adequate case definition	Representativeness of cases	Selection of controls	Definition of controls			Same method of ascertainment		
El Maghraoui [10]	*	–	*	*	*	*	*	–	6
Kao [11]	*	*	–	*	*	*	–	–	5
Merle B [14]	*	–	*	*	*	*	*	–	6
Neto [15]	*	*	*	*	**	*	*	–	8
Sahin [16]	*	–	*	–	*	*	*	–	5
Sahin [17]	*	*	*	*	*	*	*	*	8
Yurdakul [18]	*	*	*	–	–	*	*	–	5
Røren Nordén [19]	*	*	*	*	*	*	*	*	8
Marcora [20]	*	*	*	*	**	*	*	*	9
Dos Santos [12]	*	–	*	–	*	*	*	–	5
Toussirot [13]	*	*	*	–	*	*	*	*	7

*Each asterisk represents if individual criterion within the subsection was fulfilled

*NOS* Newcastle–Ottawa quality assessment scale

### Study characteristics

Among the 14 papers retained, one [9] was a prospective study, 11 [10–20] were case–control studies and two were a cross-sectional study [21, 22]. All studies investigated axial SpA and muscle mass or strength evaluation, and met the aforementioned inclusion criteria. In detail, five investigated the presence of sarcopenia [10, 14, 15, 21, 22]; two considered only muscle mass assessment [12, 13]; five analyzed only the relationship between the rheumatic disease and muscle strength [9, 11, 16–18]. Finally, two considered both muscle mass and strength but without assessing the presence of sarcopenia [19, 20]. All articles were published between 2001 and 2023. Twelve studies focused on a comparison with a healthy population — seven of which included only male patients [10, 12, 16–20]. Overall, a total of 1233 participants were included in the studies: 596 patients had AS. Pre-sarcopenia was assessed by the Baumgartner definition [23] or by the presence of only muscle mass reduction [24]. Sarcopenia was assessed using the European Working Group on Sarcopenia in Older People (EWGSOP) criteria 2010 [24], the European Working Group on Sarcopenia in Older People (EWGSOP2) criteria 2019 [6] or the Asian Working Group for Sarcopenia (AWGS) criteria 2019 [25].

The studies were carried out in Turkey [9, 16–18], France [12–14], Morocco [10], Taiwan [11], Portugal [15], Norway [19], the U.K.[20], Italy [21] and Thailand [22].

The individual outcomes are briefly discussed below. Supplementary Table 1 lists all the selected studies highlighting the main results about any positive or negative association between axial SpA and muscle health.

### Muscle mass

Only three authors reported low appendicular mass values in AS patients [10, 19, 20]. The case–control study by El Maghraoui et al.- 2016 was conducted on 134 male individuals (67 with AS, mean age 40.7 ± 11.0 years) aiming to define the prevalence of pre-sarcopenia and sarcopenia, and to analyze its relationship with rheumatic disease parameters [10]; a significant reduction of appendicular lean mass (22.2 ± 3.0 vs. 23.4 ± 3.3, *p* = 0.033) but not appendicular mass index was reported in AS patients [10]. Two other case–control studies conducted on 10 (mean age 39 ± 4.2 years) [19] and 19 (mean age 53 ± 12) [20] male patients, respectively, reported lower appendicular lean mass values (measured by Dual-Energy X-ray Absorptiometry-DXA) when compared to healthy controls [(8.3 ± 0.9 vs. 8.8 ± 0.8 kg/m^2^, *p* = 0.02) and (21.9 ± 2.8 vs. 24.9 ± 4.2 kg), respectively] [19, 20].

The measure of total lean mass by DXA did not show any significant differences between cases and controls in the studies by Toussirot et al. [13] and Dos Santos et al. [12]; the former considered 71 patients with AS (median age 38 years) compared to as many controls [13], the latter comprised 39 male patients (37.6 ± 9.1 years) with rheumatoid disease [12]. Finally, two recent studies aiming to ascertain the prevalence of sarcopenia in AS patients described low skeletal muscle mass in these patients, with no significant differences as it relates to lean mass (total or appendicular) when compared to controls [14, 15].

### Muscle strength

The studies related to the measurement of muscle strength considered heterogeneous tools: to assess the strength of the hand (handgrip dynamometer) and the strength of the lower extremity (knee-extension device, isokinetic dynamometer). Among the studies that analyzed differences on lean mass, two also focused on muscle strength, though no significative differences were reported when handgrip dynamometer was applied [19, 20]. In the study of Marcora et al. [20], handgrip strength was 40.1 ± 8.0 kg in AS patients vs. 40.4 ± 8.9 kg in healthy controls; however, knee extensors strength was significantly lower in the rheumatic patients in both case–control studies (181 ± 67 Nm vs 228 ± 72 Nm p = 0.04 and 187 ± 38 Nm vs 226 ± 29 Nm, *p* = 0.03, respectively) [19, 20]. Similar results were obtained in the recent study by Kao et al. [11], which focused on 51 AS patients and controls (mean age 41.7 ± 11.8 years), with low handgrip dynamometer scores in affected patients vs. healthy controls (30.23 ± 11.0 vs. 38.76 ± 8.23, *p* = 0.003). The Authors underlined that there were no correlations between muscle strength and disease activity, regardless of the instrument used to evaluate it [11].

The prospective study of Demirkapi et al. [9], enlisted 60 patients (39 with AS; mean age 39.3 ± 8.6 years), with the aim to evaluate muscle performance in these patients using an isokinetic dynamometer. Peak torque values in AS patients were lower than in controls both in flexors and extensors muscles at 60°/second and 180°/second angular velocity [9].

One hundred males (50 with AS) were included in the study by Yurdakul et al. [18] to compare the muscle strength of different muscle groups in AS patients vs. healthy controls [18]: the measurements by manual muscle tester in all areas were lower in AS patients, later confirmed via analyses of relevant regions (mean values: hip, 79.72 ± 28.24 vs. 101.24 ± 21.57; shoulder, 69.48 ± 24.38 vs. 88.28 ± 23.14; cervical, 26.01 ± 10.18 vs. 33.49 ± 8.21; truncal, 30.68 ± 13.47 vs. 39.04 ± 8.96. *p* < 0.001 for all) [18]. Furthermore, there was a correlation between strength of individual muscle group and disease activity: significative results were obtained for hip internal and external rotation (mean values: r =  − 0.40 and r = − 0.41, *p* < 0.01, respectively; max values: r =  − 0.41, *p* < 0.01 and r =  − 0.34, *p* < 0.05, respectively) [18].

The two case-controls studies of Sahin et al. [16, 17] used an isokinetic dynamometer to compare strength of ankle plantarflexor/dorsiflexor muscles [16] and knee extensor/flexor muscle groups [17] in 27 AS patients (mean age 37.04 ± 8.85 years) and healthy controls. Among patients, the dynamometer measurements were lower in all angular velocities (*p* < 0.05 for all).

Finally, four studies used a hand dynamometer for sarcopenia evaluation [10, 14, 15, 21], but only two reported low muscle strength values in AS patients [14, 15]. The French study of Merle et al. [14], conducted on 206 patients (53 with axial SpA, mean age 43.6 ± 12.2) aimed to evaluate the prevalence of probable and confirmed sarcopenia in AS patients; the authors only found a significant reduction in muscle strength between cases and controls (28.8 ± 13.1 vs. 31.5 ± 6.6, *p* < 0.05), especially in women (20.8 ± 6.9 vs. 28.5 ± 4.2, *p* < 0.001) [14]. A similar study by Neto et al. [15] reported a significant reduction in strength values both in upper and lower extremity: 47.6 (40.2–73.2) vs. 71.8 (51.9–80.5) and 51.0 (38.5–57.1) vs. 59.8 (54.6–64.5), respectively. Moreover, a reduction in muscle strength was observed in 8.3% of AS patients vs. 0% of controls [15]. The study was conducted on 54 patients (27 with AS and 27 healthy controls), mean age 36.5 ± 7.5 years, strength was assessed through a resisted hand-held dynamometer [15]. However, sarcopenic AS patients showed no differences in handgrip strength compared AS patients without sarcopenia (19.5 ± 7.0 vs. 25.3 ± 9.8) in the study by El Maghraoui et al. [10].

### Sarcopenia

Of the five studies that focused on sarcopenia [10, 14, 15, 21, 22], three considered the most recent guidelines for the diagnosis of this musculoskeletal disease [14, 15, 22]. The French study by Merle et al. 2023, reported a significant percentage of patients with probable sarcopenia (21% vs 7% in controls, *p* < 0.01), i.e. reduction of grip strength [14]. Although the authors did not find a direct relationship between sarcopenia and rheumatic disease activity, they proposed a questionnaire (SarQoL) to assess patients’ perception of physical, psychological and social quality of life, finding that patients with lower grip strength scores also had lower SarQoL scores (44.3 ± 11.6 vs. 61.0 ± 15.8, *p* < 0.001) [14]. Similarly, no sarcopenic patients were detected in the case–control study by Neto et al. [15]: a reduction in skeletal muscle mass and strength (8.3% vs. 4.2% and 8.3% vs. 0% of the sample, respectively) was reported in young AS patients, albeit without ever reaching statistical significance [15].

A recent study from Thailand considered the Asian Working Group for Sarcopenia 2019 guidelines for the diagnosis of sarcopenia [22]. The authors endeavored to evaluate sarcopenia in a cohort of 104 patients (mean age 42 years) diagnosed with AS. Among these, 26 individuals received biologics and anti-TNF agents were the most frequently prescribed [22]. Sarcopenia was identified in 89 subjects (85.6%), with impaired physical performance presence in 23 (22.1%). Sarcopenic patients exhibited older age and lower BMI compared to their non-sarcopenic counterparts, with no discernible differences in sex, disease duration, activity or severity, and use of biologics and glucocorticoids [22]. Nevertheless, sarcopenic patients exhibited a higher incidence of osteoporosis compared to their non-sarcopenic counterparts, with 7 subjects (6.7%) diagnosed with osteosarcopenia [22]. In multivariate analyses, older age, lower BMI, and higher BASFI were identified as independent factors associated with sarcopenia in patients with AS (22).

Both studies of Barone et al. [21] and of El Maghraoui et al. [10] distinguished between pre-sarcopenia and sarcopenia. The former was a cross-sectional study comprising 168 patients with different rheumatic diseases (22 with AS, mean age 51.6 ± 8.8 years) [21]. The authors found no significant differences among groups as it pertains to the presence of pre-sarcopenia or sarcopenia, and the results were confirmed by a logistic regression analysis; notably, there were higher rates of pre-sarcopenia in the AS group (36.6% vs. 25.7% in psoriatic arthritis and 10.5% in rheumatoid arthritis, p = 0.006) [21]. About 50% of patients with sarcopenia also had active SpA (r_phi_ − 0.55, r^2^ 0.30) [21]. In the study by El Maghraoui et al. [10], a significative reduction in appendicular lean mass (22.2 ± 3.0 vs. 23.4 ± 3.3, *p* = 0.033) but no of appendicular mass index neither muscle strength was reported in AS patients [10]. About half of patients with AS had pre-sarcopenia (50.7% vs. 28%, *p* < 0.01), whereas 34% had sarcopenia [10]. Finally, SpA patients with pre-sarcopenia and sarcopenia had higher BASDAI scores (4.4 ± 2.4 vs. 3.3 ± 2.5 and 4.4 ± 2.4 vs. 3.4 ± 2.5, *p* = 0.003 respectively), suggesting active disease. A multiple regression analysis revealed that active disease score was the only variable associated with pre-sarcopenia, defined as a reduction in skeletal mass index (OR 1.050, IC95% 1.002–1.086, *p* = 0.03) [10].

## Discussion

Sarcopenia is typically described as a condition of the aging process. There is growing interest in the secondary forms of sarcopenia, linked to immobilization/bed rest, osteoporosis and chronic inflammation, typical of rheumatic diseases [26, 27]. This comprehensive systematic review included 14 articles encompassing a population of 596 patients affected by AS. Our review aimed to assess the prevalence of pre-sarcopenia, probable sarcopenia, and sarcopenia in patients with AS. Additionally, we aimed to separately examine two pivotal aspects of muscle health, namely muscle mass and muscle strength. This approach stems from the recognition that muscle strength and muscle mass, as distinct health indicators, are regulated through different mechanisms. While the methodologies for evaluating muscle mass, strength, and sarcopenia varied among studies, the collective evidence suggests a potential correlation between compromised muscle health and AS in adults, irrespective of age.

AS can manifest as systemic fatigue and generalized stiffness; however, the primary impairment is notably observed in the pelvic, lumbar, dorsal, and cervical muscles, contingent on the extent of disease involvement. Despite the limited examination of trunk muscles' function in a singular study [14], a noteworthy decline in muscle strength was identified, suggesting that muscle strength might serve as a significant symptom in SpA [14]. This observation warrants further investigation for more comprehensive characterization.

**Fig. 2:**
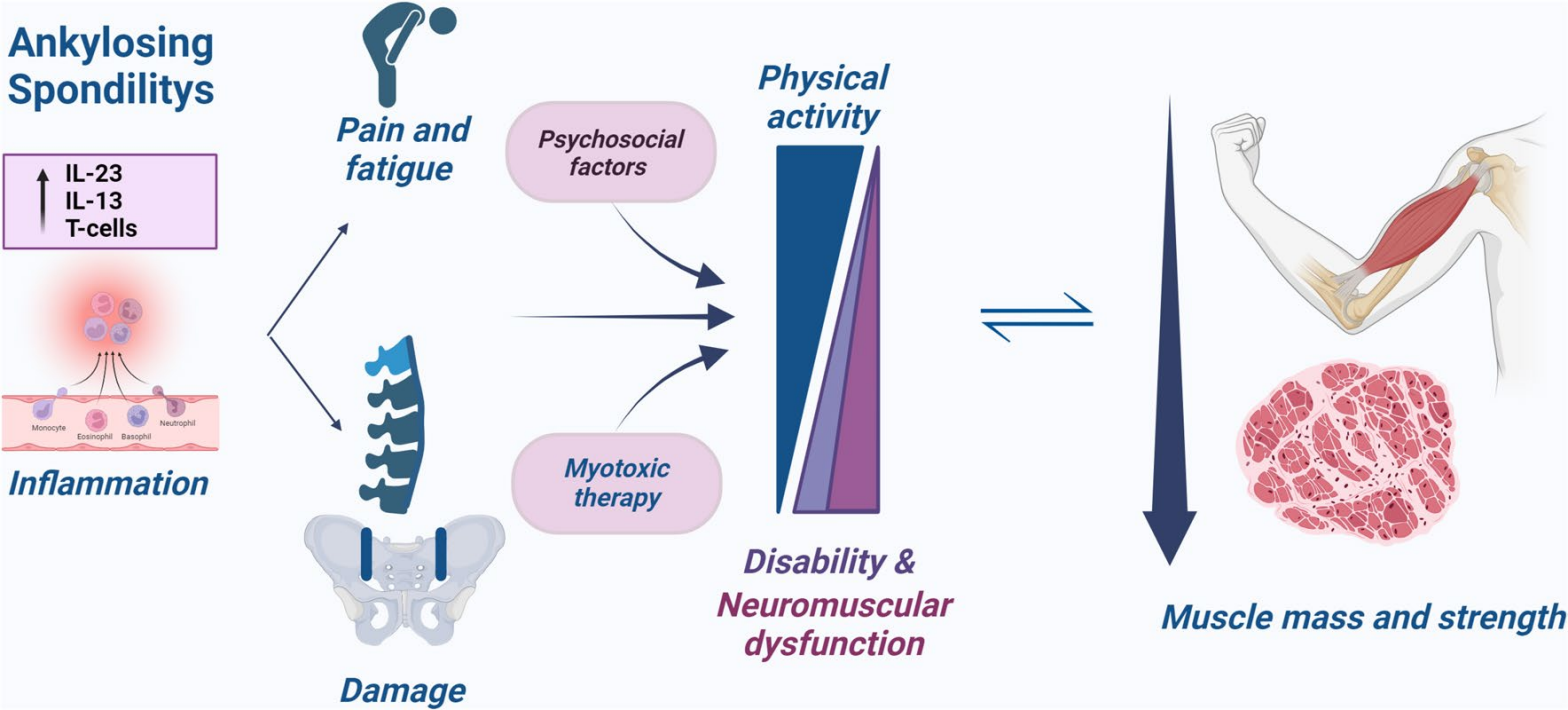
Potential mechanisms in the association between Ankylosing Spondylitis and muscle health. Inflammation and damage, together with psychosocial factors, are the main determinants of the impact of ankylosing spondylitis on physical function. A decreased physical activity pairs with the increase of disability and assistential needs. Physical inactivity and progressive neuromuscular dysfunction lead to muscle mass wasting and strength decline, in a vicious circle ultimately leading to pre-sarcopenia and sarcopenia. In some cases, external contributors to muscular impairment could be myotoxic agents such as glucocorticoids, that are known to reduce muscle metabolism and impair contractile mechanisms

While the specific mechanisms contributing to muscle damage are still debated, our hypothesis suggests that inflammation, reduced physical activity, a sedentary lifestyle, glucocorticoid therapy, and neuromuscular impairment may all play integral roles (Fig. 2). In the context of sarcopenia development, inflammation assumes a crucial role [28–30]. Elevated inflammation and disease activity may lead to sarcopenia, compromising muscle performance, reducing quality of life, and contributing to the overall burden of the disease [28]. The pathogenesis of ankylosing spondylitis involves a complex interplay of genetic and environmental factors. Aberrantly stimulated innate and innate-like cells, such as γδ T cells, group 3 innate lymphoid cells, and mucosa-associated invariant T cells, activate the IL-23/-17 axis, resulting in a local pro-inflammatory environment [31, 32]. In turn, inflammatory cytokines activate various molecular pathways associated with skeletal muscle wasting, creating an imbalance between protein synthesis and catabolism. Moreover, a recent literature review has highlighted how muscle depletion and sarcopenia are closely linked to neuromuscular degradation, a process quantifiable through the assessment of biomarkers indicating neuromuscular junction (NMJ) stability, such as the C-terminal agrin fragment (CAF) [33]. The presence of CAF in the bloodstream correlates with NMJ decline and muscle denervation, showing a significant association with muscle mass loss [33]. Consequently, CAF serves as an early indicator of sarcopenia, both in its primary and secondary forms [33].

It's noteworthy that the participants in these studies were generally young, with four studies involving biological drug treatments [8–10, 13], and in another four studies, BASDAI values were notably low [14–16, 18]. This factor may partially account for the lower level of significance observed in some results. Nevertheless, the majority of studies consistently revealed lower muscle mass and/or muscle strength in the AS population compared to controls, indicating residual muscle dysfunction even in a state of low disease activity. Another significant contributor to muscle damage is physical inactivity [34]. The onset of the disease and its exacerbations are associated with reduced activity, fatigue, and progressive impairments, leading to disability and the need for assistance in even the simplest daily tasks [35]. Additionally, neuromuscular impairment—manifested as local inflammation and damage to tendons and ligaments—may contribute to pre-sarcopenic changes in patients with AS, resulting in a loss of coordination of voluntary muscle movement [36].

Hence, non-pharmacological interventions such as physical exercise are crucial for maintaining and improving neuromuscular function, mobility, and functional capacity, ultimately reducing pain and preventing joint deformity [37, 38]. Previous studies have highlighted an association between disease activity, a sedentary lifestyle, and poor quality of life, serving as independent risk factors for comorbidities like sarcopenia in patients with AS. Conversely, physical activity is linked to better function, exercise capacity, and spinal mobility [38–40]. This underscores the importance of a multidisciplinary approach in managing AS patients, involving close collaboration between healthcare professionals such as physiatrists and physiotherapists. These professionals play a crucial role in facilitating the functional recovery of patients, thereby improving their quality of life and mitigating complications [41].

Initiating physical therapy, which includes kinesiotherapy and rehabilitation programs involving exercises and mind–body techniques, early in the course of AS is imperative and should be an integral aspect of disease management [41–43]. Physical exercises can be undertaken independently or under supervision [41]. The latter entails the instruction and demonstration of exercises, coupled with open discussions about feelings and concerns with the physiotherapist, fostering a trusting relationship that may enhance patients' adherence to physiotherapy programs [44]. A recent meta-analysis has demonstrated that supervised physiotherapy is comparable to home-based exercises but more effective than usual care in ameliorating disease activity, enhancing functional capacity, and alleviating pain in patients with AS [44]. Notably, exercises such as Pilates, aquatic exercises, aerobic and stretching exercises, ultrasound therapies, cardiovascular training, and Baduanjin Qigong exercise were considered in this context [44]. Furthermore, cryotherapy and kinesiotherapy yielded promising results in AS patients, with the BASDAI index decreasing by approximately 40% compared to baseline in 32 patients undergoing whole-body cryotherapy followed by kinesiotherapy, as opposed to 16 patients treated with kinesiotherapy alone [45]. However, as of now, there is no specific non-pharmacological protocol, and clear indications regarding which exercises are most beneficial to patients remain elusive [41, 44].

A crucial aspect in comprehending the pathogenesis of sarcopenia in patients with AS is the use of drugs for pain management. Pain, considered the most important patient-reported outcome (PRO) in rheumatology, significantly impacts health-related quality of life [46]. Commonly used medications for pain management and inflammation in AS include non-steroidal anti-inflammatory drugs (NSAIDs), glucocorticoids, opioids, and neuromodulators [46]. Glucocorticoids have an immediate detrimental effect on muscle health, altering sarcolemmal excitability, protein synthesis, and myogenesis, ultimately reducing muscle strength [7, 47]. While a recent systematic literature review reported that biologic disease-modifying antirheumatic drugs (bDMARDs) play a role in improving muscle mass and strength in SpA patients, their impact on total lean mass remains insignificant [7]. However, our results do not provide clarity on whether the damage induced by disease activity and glucocorticoids can be fully reversed by bDMARDs alone. Our impression aligns with the suggestion in the 2022 ASAS-EULAR guidelines that bDMARDs therapy alone may not be sufficient for the complete restoration of healthy muscle [41].

### Limitations

We would be remiss not to mention some of the limitations of our review. Firstly, the lack of longitudinal studies that uniformly assessed strength deficit and loss of muscle mass; cross-sectional studies are not sufficient to evaluate the real impact of sarcopenia as a suspected consequence of AS. Secondly, although most AS patients are commonly treated with bDMARDs nowadays, we did not evaluate the effects of these drugs on muscle health as it was beyond the scope of our study. Furthermore, we did not consider severe sarcopenia, instead focusing solely on muscle mass and strength parameters. Another limitation is that only two studies considered the latest guidelines to ascertain the presence of sarcopenia, thus making the results regarding its prevalence in patients with AS very heterogeneous. Moreover, these definitions are usually applied to older people, while AS predominantly affects young adults. The studies we retrieved were mostly carried out in higher-income countries, which are not representative of the world’s general population. Only one study was conducted in low- and middle-income countries (LMIC) [22], which may limit the applicability of our findings.

## Conclusions

In conclusion, our systematic review found evidence that muscle damage, especially reduced power and strength, are associated with AS. These patients may benefit from early and targeted interventions to improve muscle health and therefore quality of life. We hope that our work can contribute to reflecting on an emerging issue for these young patients, which has the potential to be prevented and treated in order to ensure successful aging for them. Larger prospective studies are warranted to better understand the pathophysiological mechanisms and the impact of other risk factors for sarcopenia.

## Supplementary Information

Below is the link to the electronic supplementary material.

Supplementary file1 (DOCX 22 kb)

## References

[1] Baeten D, Breban M, Lories R, Schett G, Sieper J (2013) Are spondylarthritides related but distinct conditions or a single disease with a heterogeneous phenotype? Arthritis Rheum 65(1):12–20 23288559 10.1002/art.37829

[2] Sepriano A, Ramiro S, van der Heijde D, van Gaalen F, Hoonhout P, Molto A et al (2020) What is axial spondyloarthritis? A latent class and transition analysis in the SPACE and DESIR cohorts. Ann Rheum Dis 79(3):324–331 31980546 10.1136/annrheumdis-2019-216516

[3] Mauro D, Gandolfo S, Tirri E, Schett G, Maksymowych WP, Ciccia F (2023) The bone marrow side of axial spondyloarthritis. Nat Rev Rheumatol 19(8):519–532 37407716 10.1038/s41584-023-00986-6

[4] Mei J, Hu H, Ding H, Huang Y, Zhang W, Chen X et al (2023) Investigating the causal relationship between ankylosing spondylitis and osteoporosis in the European population: a bidirectional Mendelian randomization study. Front Immunol 14:1163258 37359532 10.3389/fimmu.2023.1163258 PMC10285397

[5] An HJ, Tizaoui K, Terrazzino S, Cargnin S, Lee KH, Nam SW, et al. (2020) Sarcopenia in autoimmune and rheumatic diseases: a comprehensive review. Int J Mol Sci 21(16) 10.3390/ijms21165678 PMC7461030 32784808

[6] Cruz-Jentoft AJ, Bahat G, Bauer J, Boirie Y, Bruyère O, Cederholm T et al (2019) Sarcopenia: revised European consensus on definition and diagnosis. Age Ageing 48(1):16–31 30312372 10.1093/ageing/afy169 PMC6322506

[7] Ben Tekaya A, Mehmli T, Ben Sassi M, Teyeb Z, Bouden S, Rouached L et al (2023) Effects of biologic and target synthetic disease-modifying anti-rheumatic drugs on sarcopenia in spondyloarthritis and rheumatoid arthritis: a systematic review and meta-analysis. Clin Rheumatol 42(4):979–997 36462127 10.1007/s10067-022-06454-y

[8] Stroup DF, Berlin JA, Morton SC, Olkin I, Williamson GD, Rennie D et al (2000) Meta-analysis of observational studies in epidemiology: a proposal for reporting. Meta-analysis of observational studies in epidemiology (MOOSE) group. JAMA 283(15):2008–2012 10789670 10.1001/jama.283.15.2008

[9] Demirkapi M, Yildizgören MT, Güler H, Turhanoğlu AD (2017) The effect of anti-tumor necrosis factor-alpha treatment on muscle performance and endurance in patients with ankylosing spondylitis: a prospective follow-up study. Arch Rheumatol 32(4):309–314 29901011 10.5606/ArchRheumatol.2017.6335 PMC5868461

[10] El Maghraoui A, Ebo’o FB, Sadni S, Majjad A, Hamza T, Mounach A (2016) Is there a relation between pre-sarcopenia, sarcopenia, cachexia and osteoporosis in patients with ankylosing spondylitis? BMC Musculoskelet Disord 17(1):1–8 27401188 10.1186/s12891-016-1155-z PMC4940725

[11] Kao CI, Liau BY, Lai KL, Kuo FC (2023) Correlation among disease activity, musculoskeletal function, and quality of life in patients with ankylosing spondylitis with mild to moderate radiographic signs. J Med Biol Eng 43(2):147–155 10.1007/s40846-023-00780-5

[12] Dos Santos FP, Constantin A, Laroche M, Destombes F, Bernard J, Mazières B et al (2001) Whole body and regional bone mineral density in ankylosing spondylitis. J Rheumatol 28(3):547–549 11296956

[13] Toussirot E, Michel F, Wendling D (2001) Bone density, ultrasound measurements and body composition in early ankylosing spondylitis. Rheumatology 40(8):882–888 11511757 10.1093/rheumatology/40.8.882

[14] Merle B, Cottard M, Sornay-Rendu E, Szulc P, Chapurlat R (2023) Spondyloarthritis and sarcopenia: prevalence of probable sarcopenia and its impact on disease burden: the saspar study. Calcif Tissue Int 112(6):647–655 36944706 10.1007/s00223-023-01074-3

[15] Neto A, Pinheiro Torres R, Ramiro S, Sardoo A, Rodrigues-Manica S, Lagoas-Gomes J et al (2022) Muscle dysfunction in axial spondylarthritis: the MyoSpA study. Clin Exp Rheumatol 40(2):267–273 34874829 10.55563/clinexprheumatol/9ljng7

[16] Sahin N, Ozcan E, Baskent A, Karan A, Ekmeci O, Kasikcioglu E (2011) Isokinetic evaluation of ankle muscle strength and fatigue in patients with ankylosing spondylitis. Eur J Phys Rehabil Med 47(3):399–405 21364512

[17] Sahin N, Ozcan E, Baskent A, Karan A, Kasikcioglu E (2011) Muscular kinetics and fatigue evaluation of knee using by isokinetic dynamometer in patients with ankylosing spondylitis. Acta Reumatol Port 36(3):252–259 22113600

[18] Yurdakul OV, Ince OE, Bagcier F, Kara M, Kultur E, Aydin T (2021) Evaluating the strength of spinal and proximal girdle muscles in patients with axial spondyloarthritis: Correlation with activity, disability, and functionality. Int J Rheum Dis 24(5):701–710 33750032 10.1111/1756-185X.14102

[19] Røren Nordén K, Dagfinrud H, Løvstad A, Raastad T (2016) Reduced appendicular lean body mass, muscle strength, and size of type II muscle fibers in patients with spondyloarthritis versus healthy controls: a cross-sectional study. ScientificWorldJournal 2016:6507692 27672678 10.1155/2016/6507692 PMC5031855

[20] Marcora S, Casanova F, Williams E, Jones J, Elamanchi R, Lemmey A (2006) Preliminary evidence for cachexia in patients with well-established ankylosing spondylitis. Rheumatology 45(11):1385–1388 16603581 10.1093/rheumatology/kel127

[21] Barone M, Viggiani M, Anelli M, Fanizzi R, Lorusso O, Lopalco G et al (2018) Sarcopenia in patients with rheumatic diseases: prevalence and associated risk factors. J Clin Med 7(12):504 30513782 10.3390/jcm7120504 PMC6306844

[22] Kanjanavaikoon N, Saisirivechakun P, Chaiamnuay S (2023) Age, body mass index, and function as the independent predictors of sarcopenia in axial spondyloarthritis: a cross-sectional analysis. Clin Rheumatol 42(12):3257–3265 37755546 10.1007/s10067-023-06770-x

[23] Baumgartner RN, Koehler KM, Gallagher D, Romero L, Heymsfield SB, Ross RR, et al (1998) Epidemiology of sarcopenia among the elderly in New Mexico. Am J Epidemiol 10.1093/oxfordjournals.aje.a009520 9554417

[24] Cruz-Jentoft AJ, Baeyens JP, Bauer JM, Boirie Y, Cederholm T, Landi F et al (2010) Sarcopenia: European consensus on definition and diagnosis: report of the European Working Group on Sarcopenia in older people. Age Ageing 39(4):412–423 20392703 10.1093/ageing/afq034 PMC2886201

[25] Chen L-K, Woo J, Assantachai P, Auyeung T-W, Chou M-Y, Iijima K et al (2020) Asian Working Group for Sarcopenia: 2019 consensus update on sarcopenia diagnosis and treatment. J Am Med Dir Assoc 21(3):300-307.e2 32033882 10.1016/j.jamda.2019.12.012

[26] Salaffi F, Di Matteo A, Farah S, Di Carlo M (2023) Inflammaging and frailty in immune-mediated rheumatic diseases: how to address and score the Issue. Clin Rev Allergy Immunol 64(2):206–221 35596881 10.1007/s12016-022-08943-z PMC10017626

[27] Aguiar R, Sequeira J, Meirinhos T, Ambrósio C, Barcelos A (2014) SARCOSPA-sarcopenia in spondyloarthritis patients. Acta Reumatol Port 2014(4):322–326 25584619

[28] Valido A, Crespo CL, Pimentel-Santos FM (2019) Muscle evaluation in axial spondyloarthritis-the evidence for sarcopenia. Front Med 6:219 10.3389/fmed.2019.00219 PMC6813235 31681777

[29] Bano G, Trevisan C, Carraro S, Solmi M, Luchini C, Stubbs B et al (2017) Inflammation and sarcopenia: A systematic review and meta-analysis. Maturitas 96:10–15 28041587 10.1016/j.maturitas.2016.11.006

[30] Lorenzin M, Ometto F, Ortolan A, Felicetti M, Favero M, Doria A et al (2020) An update on serum biomarkers to assess axial spondyloarthritis and to guide treatment decision. Ther Adv Musculoskelet Dis 12:1759720X20934277 32636944 10.1177/1759720X20934277 PMC7315656

[31] Mauro D, Thomas R, Guggino G, Lories R, Brown MA, Ciccia F (2021) Ankylosing spondylitis: an autoimmune or autoinflammatory disease? Nat Rev Rheumatol 17(7):387–404 34113018 10.1038/s41584-021-00625-y

[32] Cozzi G, Scagnellato L, Lorenzin M, Savarino E, Zingone F, Ometto F et al (2023) Spondyloarthritis with inflammatory bowel disease: the latest on biologic and targeted therapies. Nat Rev Rheumatol 19(8):503–518 37386288 10.1038/s41584-023-00984-8

[33] Kumar P, Nayak K, Umakanth S, Girish N (2023) Effect of targeted intervention on C-terminal agrin fragment and its association with the components of sarcopenia: a scoping review. Aging Clin Exp Res 35(6):1161–1186. 10.1007/s40520-023-02396-w 36977974 10.1007/s40520-023-02396-w PMC10200783

[34] Ramonda R, Marchesoni A, Carletto A, Bianchi G, Cutolo M, Ferraccioli G et al (2016) Patient-reported impact of spondyloarthritis on work disability and working life: the ATLANTIS survey. Arthritis Res Ther 18:78 27037139 10.1186/s13075-016-0977-2 PMC4818386

[35] de Hooge M, Ramonda R, Lorenzin M, Frallonardo P, Punzi L, Ortolan A et al (2016) Work productivity is associated with disease activity and functional ability in Italian patients with early axial spondyloarthritis: an observational study from the SPACE cohort. Arthritis Res Ther 18(1):265 27852321 10.1186/s13075-016-1162-3 PMC5112652

[36] Moreira-Pais A, Ferreira R, Oliveira PA, Duarte JA (2022) A neuromuscular perspective of sarcopenia pathogenesis: deciphering the signaling pathways involved. GeroScience 44(3):1199–1213 34981273 10.1007/s11357-021-00510-2 PMC9213593

[37] Lim H-J, Lee MS, Lim H-S (2005) Exercise, pain, perceived family support, and quality of life in Korean patients with ankylosing spondylitis. Psychol Rep 96(1):3–8 15825897 10.2466/pr0.96.1.3-8

[38] Coulter EH, McDonald MT, Cameron S, Siebert S, Paul L (2020) Physical activity and sedentary behaviour and their associations with clinical measures in axial spondyloarthritis. Rheumatol Int 40(3):375–381 31848736 10.1007/s00296-019-04494-3 PMC7002460

[39] Masiero S, Bonaldo L, Pigatto M, Lo Nigro A, Ramonda R, Punzi L (2011) Rehabilitation treatment in patients with ankylosing spondylitis stabilized with tumor necrosis factor inhibitor therapy: a randomized controlled trial. J Rheumatol 38(7):1335–1342 21459942 10.3899/jrheum.100987

[40] Masiero S, Poli P, Bonaldo L, Pigatto M, Ramonda R, Lubrano E et al (2014) Supervised training and home-based rehabilitation in patients with stabilized ankylosing spondylitis on TNF inhibitor treatment: a controlled clinical trial with a 12-month follow-up. Clin Rehabil 28(6):562–572 24285801 10.1177/0269215513512214

[41] Ramiro S, Nikiphorou E, Sepriano A, Ortolan A, Webers C, Baraliakos X et al (2023) ASAS-EULAR recommendations for the management of axial spondyloarthritis: 2022 update. Ann Rheum Dis 82(1):19–34 36270658 10.1136/ard-2022-223296

[42] Huang D, Ke X, Jiang C, Song W, Feng J, Zhou H et al (2023) Effects of 12 weeks of Tai Chi on neuromuscular responses and postural control in elderly patients with sarcopenia: a randomized controlled trial. Front Neurol 14:1167957 37188307 10.3389/fneur.2023.1167957 PMC10176447

[43] Ortolan A, Webers C, Sepriano A, Falzon L, Baraliakos X, Landewé RB et al (2023) Efficacy and safety of non-pharmacological and non-biological interventions: a systematic literature review informing the 2022 update of the ASAS/EULAR recommendations for the management of axial spondyloarthritis. Ann Rheum Dis 82(1):142–152 36261247 10.1136/ard-2022-223297

[44] Gravaldi LP, Bonetti F, Lezzerini S, De Maio F (2022) Effectiveness of physiotherapy in patients with ankylosing spondylitis: a systematic review and meta-analysis. Healthc 10(1) 10.3390/healthcare10010132 PMC8775656 35052296

[45] Stanek A, Cholewka A, Gadula J, Drzazga Z, Sieron A, Sieron-Stoltny K (2015) Can whole-body cryotherapy with subsequent kinesiotherapy procedures in closed type cryogenic chamber improve BASDAI, BASFI, and some spine mobility parameters and decrease pain intensity in patients with ankylosing spondylitis? Biomed Res Int 2015:404259 26273618 10.1155/2015/404259 PMC4529896

[46] Hunter T, Nguyen C, Birt J, Smith J, Shan M, Tan H et al (2021) Pain medication and corticosteroid use in ankylosing spondylitis, psoriatic arthritis, and rheumatoid arthritis in the united states: a retrospective observational study. Rheumatol Ther 8(3):1371–1382 34312825 10.1007/s40744-021-00344-6 PMC8380595

[47] Sato AY, Richardson D, Cregor M, Davis HM, Au ED, McAndrews K et al (2017) Glucocorticoids induce bone and muscle atrophy by tissue-specific mechanisms upstream of E3 ubiquitin ligases. Endocrinology 158(3):664–677 28359087 10.1210/en.2016-1779 PMC5460781

