# Supplementary material for: Is sarcopenia a real concern in ankylosing spondylitis? A systematic literature review
Source: Eur Geriatr Med. 2024 Apr 3;15(4):903–12. doi: 10.1007/s41999-024-00968-1 (PMC11377609; doi:10.1007/s41999-024-00968-1)
Supplement: Supplementary file 1 — Supplementary file1 (DOCX 22 kb) [file 41999_2024_968_MOESM1_ESM.docx]

**Complete search strategy.**

((“ankylosing spondylitis” OR “Spondylitis, Ankylosing” OR “spondylitis” OR “spondyloarthropath*” OR “spondylarthropath*” OR “Axial Spondyloarthritis” OR “spondylitis ankylos*” OR axSPA OR r-axSPA OR nr-axSPA OR sacroileitis OR sacroiliitis OR spondyloarthrit* OR spondylarthrit*)) AND ((sarcopenia OR muscular atrophy OR cachexia OR Muscle Strength)) NOT ((exercise))

**Supplementary Table 1. Identified studies demonstrating associations between sarcopenia and SpA.**

| **Author/year/reference** | **Country** | **Study design** | **Sample size of investigated patients (N, %female gender)/controls** | **Mean/**  **median age (years)** | **Body composition assessment tool** | **Muscle strenght assessment tool** | **Pre-sarcopenia/**  **Sarcopenia assessment tool** | **AS duration (years)** | **Disease activity assessment tool and mean/median value** | **Outcome on muscle mass** | **Outcome on muscle strength** | **Prevalence of**  **pre-sarcopenia/**  **sarcopenia** | **Correlation with disease activity** | **Study limitation/**  **confunders** |
| --- | --- | --- | --- | --- | --- | --- | --- | --- | --- | --- | --- | --- | --- | --- |
| Dermirkapi M. et al, 2017 (9) | Turkey | Prospective study | 30 patients with AS (M/F 23/7) vs 30 healthy controls | 39.3±8.6 | - | Isokinetic test device (Humac®/NormTM Testing & Rehabilitation System) and isokinetic dynamometer. | - | 7.5±7.9 | BASDAI | - | The angular velocity peak torque values of the extensor muscles at 60°/second and 180°/second and the total work values at 180°/second angular velocity were lower in AS vs controls (p<0.05). | - | - | Current treatment with biologic drugs; BASDAI score was not reported. |
| El Maghraoui A. et al, 2016 (10) | Morocco | case-control study | 67 male patients with AS vs 67 healthy controls | 40.7±11 | BIA | Grip-A dyamometer (Takey, Kiki Kogyo, Japan) | Pre-sarcopenia: Baumgartner definition; sarcopenia: European Working Group on Sarcopenia in Older People (EWGSOP) criteria 2010 | 9.3±7.9 | BASDAI 3.7±2.4 | Appendicular mass was lower in AS patients (22.2±3.0 vs 23.4±3.3 in controls, p=0.033). | - | pre-sarcopenia rates: 50.7% in AS patients vs 28% in controls. 23 AS patients (34%) were sarcopenic. | BASDAI was the only variable significantly associated to pre-sarcopenia (OR 1.05, IC95% 1.002-1.086, p=0.03). | Current treatment with biologic drugs |
| Kao C. et al, 2023 (11) | Taiwan | case-control study | 51 patients with AS (M/F: 40/11) vs 23 healthy controls | 41.7±11.8 | - | Handgrip dyamometer (Jamar plus, Global Medical Devices, Delhi, India) | - | 3.68±2.62 | BASDAI 3.33±2.32; ASDAS-CRP; ASDAS-ESR | - | AS group had lower handgrip strength than the control group (30.23±11.0 vs 38.76±8.23, p=0.003) | - | No correlation between muscle strength and BASDAI, ASDAS-CRP, ASDAS-ESR. | Current treatment with biologic drugs; subgroups in AS patients according to X-ray findings. |
| Dos Santos FP et al, 2001 (12) | France | case-control study | 39 male patients with AS vs 39 healthy controls. | 37.6±9.1 | DXA using a Lunar DPX (Lunar Radiation, Madison, WI, USA) | - | - | 8.4±6.3 | BASDAI | No difference in lean mass was observed between groups. | - | - | - | BASDAI mean values were not reported; young people for the EWGSOP2 criteria application. |
| Toussirot E et al, 2001 (13) | France | case-control study | 71 patients with AS (M/F: 49/22) vs 71 healthy controls. | 38 (20-67) | DXA using a Lunar DPX (Lunar Radiation, Madison, WI, USA) | - | - | 7 (1-29) | BASDAI  4.9 (0-9.5) | No difference in lean mass was observed between groups. | - | - | - |  |
| Merle B. et al, 2023 (14) | France | case-control study | 103 patients with SpA, 53 with AS (M/F: 28/25) vs 103 healthy controls | 43.6 ± 12.2 | DXA (Hologic Discovery A, Hologic Inc., Bedford, MA, USA) | Handgrip dynamometer (Patterson Medical®, Bolingbrook, IL, USA); in controls was used a Martin Vigorimeter. | Sarcopenia: EWGSOP2 consensus criteria. | 12.9 ± 9.8 | BASDAI 4.09±2.21 | No significant different in lean mass between AS patients and controls, but the proportion of patients with a low appendicular lean mass was higher in AS (15%) than in controls (7%). | Grip strength was lower in AS (28.8 ± 13.1 vs 31.5 ± 6.6, p<0,05), especially in women (20.8 ± 6.9 vs 28.5 ± 4.2, p<0,001). | Low grip strength (probable sarcopenia): 21% in AS vs 7% in controls (p<0,01); sarcopenia: 5% in AS vs 2% in controls (NS) | Patients with lower scores of grip strength had also lower scores in the perception of physical, psychological and social aspects of life. | Current treatment with biologic drugs; AS patients included people with also peripheral AS; different tools for strength assessment in case and controls. |
| Neto A. et al, 2022 (15) | Portugal | case-control study | 27 patients with axial AS (M/F: 18/9) vs 27 healthy controls | 36.5 ± 7.5 | BIA | Isometric muscle strength of trunk, upper and lower limbs, on both sides was quantified by a resisted hand-held dynamometer (Lafayette Manual Muscle Tester). | Sarcopenia: EWGSOP2 consensus criteria. | 6.5 ± 3.2 | BASDAI 3±2 | Skeletal muscle mass was reduced in other 8.3% of AS patients vs. 4.2% of controls (p=0.55). No difference in total or segmental lean mass between groups. | Low muscle strength: 8.3% AS vs 0% of controls (p=0.15); upper and lower limb strength lower in AS group: 47.6 (40.2–73.2) vs 71.8 (51.9–80.5) and 51.0 (38.5–57.1) vs 59.8 (54.6–64.5), p<0.05 respectively | No participants fulfilled the definition of sarcopenia | - | Low scores for BASDAI; young people for the EWGSOP2 criteria application. |
| Sahin N. et al, 2011 (16) | Turkey | case-control study | 26 male patients with AS vs 26 healthy controls | 37.04±8.85 | - | Isokinetic dynamometer. | - | - | BASDAI 2.28±2.0 | - | Ankle plantarflexion muscle strength was lower compared to the control group in all angular velocities (p<0.001) | - | No relation between the decreased muscle strength and pain severity | BASDAI scores were low, suggesting a low disease activity; no disease duration was reported; young people for the EWGSOP2 criteria application. |
| Sahin N. et al, 2011 (17) | Turkey | case-control study | 27 male patients with AS vs 26 healthy controls | 37.04±8.86 | - | Isokinetic dynamometer. | - | - | BASDAI 2.28±1.41 | - | Knee extension/flexion muscle strength in patient group was lower compared to the control group in all angular velocities (p< 0.05). | - | No relation between the decreased muscle strength and diasease severity | BASDAI scores were low, suggesting a low disease activity; no disease duration was reported; young people for the EWGSOP2 criteria application. |
| Kanjanavaikoon N. et al 2023 (22) | Thailand | cross-sectional study | 104 patients with AS (M/F: 74/30) | 42.6±12.6 | DXA GE-Lunar iDXA (#210754) and GE-Lunar DPX Duo densitometer (GE Healthcare, Madison, WI, USA) | Digital hand dynamometer | Sarcopenia: AWGS criteria 2019 | 8.3±8.5 | BASDAI  3.1±2.3 | - | - | Sarcopenia was identified in 89 individuals (85.6% of patients), with confirmed presence in 23 individuals (22.1%). | Low functional ability was identified as independent factors associated with sarcopenia. | There were no sex-aged, matched controls. |
| Yurdakul OV et al., 2021 (18) | Turkey | case-control study | 50 male patients with AS vs 50 healthy controls. | 39.88±11.85 | - | Manual muscle tester (Lafayette Instrument Company, Lafayette, IN, USA). | - | 57.47±90.73 | BASDAI 4.24±2.22 | - | All muscle strengths were lower in the AS group. A significant decrease of total muscle strengths was found in the AS group compared to the control group: hip 79.72 ± 28.24 vs 101.24 ± 21.57; shoulder 69.48 ± 24.38 vs 88.28 ± 23.14; cervical 26.01 ± 10.18 vs 33.49 ± 8.21; truncal 30.68 ± 13.47 vs 39.04 ± 8.96 (p<0.01 for all). | - | BASDAI had a moderate negative relationship with mean values of hip internal and external rotation (-0.40 and -0.41, p<0.01 respectively), and a weak negative relationship with hip external rotation max (-0.34, p<0.05). | Long disease activity |
| Røren Nordén K. et al, 2016 (19) | Norway | case-control study | 6 male patients with AS vs 10 healthy controls | 39±4.1 | DXA (Lunar iDXA, GE Healthcare, Buckinghamshire, United Kingdom) | An isometric knee-extension device (GYM 2000 AS, Vikersund, Norway) was used for knee extensors | - | 10.0±7.9 | BASDAI 2.7±1.1 | Appendicular lean mass was lower in the patient group (8.3±0.9 kg/m^2^ vs controls (8.8±0.8 kg/m^2^, p= 0.02). | The AS patients had lower values for maximal voluntary contraction torque vs controls (187±38 Nm vs 226 ± 29 Nm, p = 0.03) | - | - | Researches were not blinded with influence on the outcome variables. BASDAI scores were low, suggesting a low disease activity; young people for the EWGSOP2 criteria application. |
| Marcora S. et al, 2006 (20) | UK | case-control study | 19 male patients with AS vs 19 healthy controls. | 53±12 | DXA (QDR 1500, software version V5.72, Hologic Inc., Bedford MA) | An isokinetic dynamometer (Kin-com, Chattanooga, Tennessee USA) was used for knee extension, the grip-A dynamometer (Takey, Kiki Kogyo, Japan) for hand | - | 19±13 | BASDAI 4.6±2.1 | Total and appendicular lean mass were lower in AS patients (51.8 ± 6.3 vs 58.2 ±7.4 and 21.9±2.8 vs 24.9±4.2, p<0.01 respectively). | Handgrip strength was no different between groups (40.1 ±8.0 kg in AS vs 40.4 ±8.9 kg in controls, p = 0.905); knee extensors strength was lower in AS patients (181 ± 67 Nm vs 228±72 Nm, p=0.040) | - | - | Current treatment with biologic drugs |
| Barone M. et al, 2018 (21) | Italy | cross-sectional study | 22 patients with AS (M/F: 14/8) vs 76 patients with Rheumatoid Arthritis (RA) and 70 patients with Psoriatic Arthritis (PsA) | 51.6±8.8 | BIA | Handgrip dyamometer (Jamar, Sammons Preston, Bolingbrook, IL, USA) | Pre-sarcopenia: muscle mass reduction alone, without muscle strength impairment; Sarcopenia: European Working Group on Sarcopenia in Older People (EWGSOP) criteria 2010. | 14.5 ± 8.4 | BASDAI; ASDAS-CRP; ASDAS-ESR | - | - | About 22% in AS (no difference with RA and PsA). Prevalence of pre-sarcopenia was 36.6% vs 10.5% RA and 25.7% PsA. The disease type was not associated with sarcopenia at the univariate and multivariate analysis. | Patients with active disease were 50% sarcopenic and 50% non-sarcopenic (p = 0.001, by chi-square, r_phi_ = 0.55; r^2^ = 0.30). | Current treatment with biologic drugs; no healthy control population |

*Abbreviations*: SpA = spondyloarthritis; AS = Ankylosing Spondylitis; DXA = Dual-Energy X-ray Absorptiometry; BIA = Bio-Impedance Analysis; BASDAI = Bath Ankylosing Spondylitis Disease Activity Index; ASDAS-CRP = Ankylosing Spondylitis Disease Activity Score-C-reactive protein; ASDAS-ESR = Ankylosing Spondylitis Disease Activity Score-erythrocyte sedimentation rate.
